# Validation of an Age-Appropriate Screening Tool for Female Athlete Triad and Relative Energy Deficiency in Sport in Young Athletes

**DOI:** 10.7759/cureus.8579

**Published:** 2020-06-12

**Authors:** Cassidy M Foley Davelaar, Megan Ostrom, Justine Schulz, Katelyn Trane, Amy Wolkin, Julie Granger

**Affiliations:** 1 Orthopedics, Nemours Children's Health System, Orlando, USA; 2 Division of Physical Therapy, Department of Rehabilitation Medicine, Emory University School of Medicine, Atlanta, USA

**Keywords:** female athlete, young athlete, relative energy deficiency in sport, female athlete triad, medical screening

## Abstract

Background

The purpose of this study was to determine the concurrent validity of a newly created relative energy deficiency in sport (RED-S) specific screening tool (RST) by comparing scores with the validated pre-participation gynecological examination (PPGE). We hypothesized that the investigators would observe no significant difference between the means of the RST and the PPGE survey.

Methods

This was a crossover study of 39 female subjects who completed both the RST and the PPGE. The survey order was randomized.

Results

The RST was validated compared with the PPGE (Pearson’s *r* = 0.697, *p* < 0.001).

Conclusion

The administration of an RST to middle- and high-school female athletes was validated compared with the PPGE. Formatting limitations of the screening tool were highlighted, leading to changes that improved the accuracy of the screening tool prior to application in a clinical setting. The RST is an age-appropriate screening tool that can be used by coaches, athletic trainers, physical therapists, and other healthcare practitioners to detect RED-S risk and allow for earlier intervention.

## Introduction

Upon the enactment of the Educational Amendment Act (Title IX) in 1972 and the banning of sex discrimination in federally funded education programs, female athletic participation across all age ranges has increased tremendously. Female high-school athletic and intercollegiate sports participation has increased by over 900% and 450% across the country, respectively [1]. By 2009, 41% of high-school athletes were female [2]. Secondary to the increase in female athletic participation, there has been a growing concern among healthcare professionals regarding the interplay of metabolic and endocrine complexities and comorbidities pertaining to female athletes [1]. Over the past two decades, the literature has illustrated many correlates showing association between increased sports participation, overuse injury, and multiple systemic health factors in young, female athletes [3]. Health professionals are striving to identify and address these impairments, particularly through prevention. 

In 1992, the American College of Sports Medicine first termed the female athlete triad (the Triad), recognizing the interrelation of eating disorders, amenorrhea, and osteoporosis [3-7]. The terminology for the triad has continued to evolve over the years and was formally redefined in 2007 to include a broader spectrum, using newer terminology and concepts pertaining to energy availability, menstrual function, and low bone mineral density [3-7]. In 2014, the International Olympic Committee developed new terminology, which includes multisystem effects of energy deficiency that span beyond bone health and menstrual dysfunction. The new term introduced to describe multisystem dysfunction is relative energy deficiency in sport (RED-S) [3]. There is an ongoing debate as to the nomenclature and the authors are extremely respectful of the research that has brought this important issue affecting female athletes to light. They are passionate about improving the identification of this disorder, which may be described in several ways. 

The International Olympic Committee recognized that dysfunctional systemic correlates of energy deficiency were unique to female athletes, but also played a role in the health of male athletes [3]. These multisystem effects include but are not be limited to, endocrine, cardiovascular, psychological, metabolic, and immune system health. Additional implications include protein synthesis and endothelial dysfunction. The underlying cause of the triad and RED-S is low energy availability. The consequences of low energy availability are noted in male and female athletes and affect athletic performance as well as the general health of these athletes. The energy in the form of calories from dietary intake must be sufficient to support health and bodily functions after the cost of exercise has been taken into account. 

Few practitioners routinely screen for signs and symptoms of RED-S or the Triad in their injured patients [8]. In addition, there is a poor understanding of the Triad and/or RED-S in the community among athletes, coaches, and school health officials, such as school athletic trainers and nurses [9-13]. A previous cross-sectional study demonstrated that there is poor awareness among adolescent athletes regarding the connection between menstrual function and bone health [10]. The goal of this study was to create a screening tool, the RED-S specific screening tool (RST), which could be used by the general public to identify and spread awareness of the effects of low energy availability. The hypothesis of this study was that there would be no significant difference between the ability to identify low energy availability of the RST created and the Triad-specific tool, therefore validating the use of the RST. Additionally, the researchers hypothesized that there would be no significant difference between the means based on the order in which the two tests were administered.

## Materials and methods

Based on a literature review of the multifactorial components of RED-S, a pilot screening tool, the RST, was created. Due to the complexity of the syndrome, the screening tool included components of the Pre-Participation Gynecological Examination (PPGE) and an eating disorder screen (EDS) from the National Eating Disorder Association [14-15]. The PPGE is specifically for the gynecological evaluation of female athletes and not for male athletes and the EDS is for eating disorders, not unknowing low dietary intake. The aim was to include previously tested and successful screening questions into a more specific tool for a population of young male and female athletes. The PPGE was used by physicians during female pre-participation evaluations to identify when a female athlete required referral to a specialist for possible risk of developing the Triad. The components incorporated from this tool into the RST pertained to sports, injury, and menstrual history [15]. While using this tool as a reference, it is important to note that the Triad and RED-S both stem from energy availability but may present differently, so questions were only included if applicable to the cascade of RED-S. For example, components of obstetric and sexual history from the PPGE were not relevant to include on the RST and were therefore removed (Appendix A). 

The EDS is the official screening tool used by the National Eating Disorder Association to determine if professional help is recommended for eating disorders. The EDS includes questions that assist in identifying disordered eating, energy availability, bone health, metabolic rate, growth, potential dysfunctions of the gastrointestinal system, and the psychological components of RED-S [14]. Questions pertinent to the RED-S definition were sampled from this screening tool. Supplemental questions were added to address the remaining components of RED-S to fully capture the physiological and psychological aspects of RED-S. These questions covered the topics of diet, anemia, contraceptives, stress fractures, illnesses, cardiac history, and personality [3].

Because RED-S impacts both males and females, separate male and female-specific screening tools were created to capture physiological differences present between the sexes. The female screening tool not only included all questions asked within the male screening tool but also contained additional questions regarding menstruation and women’s and girls’ health. The PPGE and the EDS had previously been used in adolescent and adult populations, and thus, it was determined that the language and wording were not appropriate for the age range of the clinical study population [14-15]. The entirety of the RST’s questions was modified to a third-grade reading level, allowing younger participants to have more inclusive and age-appropriate subject matter comprehension. 

To score the RST, questions were categorized into the following components: 1) menstrual function, 2) activity levels, 3) nutrition and diet, 4) injury, 5) physiological effects, 6) psychological effects, and 7) factors that affect bone mineral density. Because energy availability is the main component of RED-S, nutrition and diet were the most highly weighted category in its contribution to risk, followed by activity levels and menstrual function. Additional categories were weighted appropriately regarding their posed risk to RED-S based on the findings of Mountjoy and colleagues [3]. Scoring depended on the participant's age. Females over the age of 16 years, and menstruating females less than 16 years of age were scored out of a total of 880 points. Males of all ages, and non-menstruating females less than 16 years of age, were scored on the same scale. These individuals could score as high as 730 points on the tool because menstrual function questions were excluded for these participants.

The RED-S risk levels were determined using the risk assessment model compiled by Mountjoy et al. [3]. Based on this model, females younger than 16 years of age and males of all ages were considered of low risk with scores less than 100, moderate risk with scores ranging from 101 to 400, and high risk with scores greater than 400. Due to the definition of primary amenorrhea, females over the age of 16 were scored on a different scale. Females 16 years of age or older were considered low risk with scores less than 150, moderate risk with scores ranging from 150 to 500, and high risk with scores greater than 500 (Appendix B).

To validate the RST, participants were recruited through convenience sampling of interscholastic public middle-school and high-school sports teams in metro Atlanta, Georgia. Inclusion criteria included both female and male athletes with the ability to read and write in English. Exclusion criteria were not applicable to this study for the purpose of convenience sampling. Subjects were included after informed consent was obtained. For children aged 11 to 17 years, assent was required from subjects as well as parental consent. For individuals older than 18 years, only informed consent was required.

**Figure 1:**
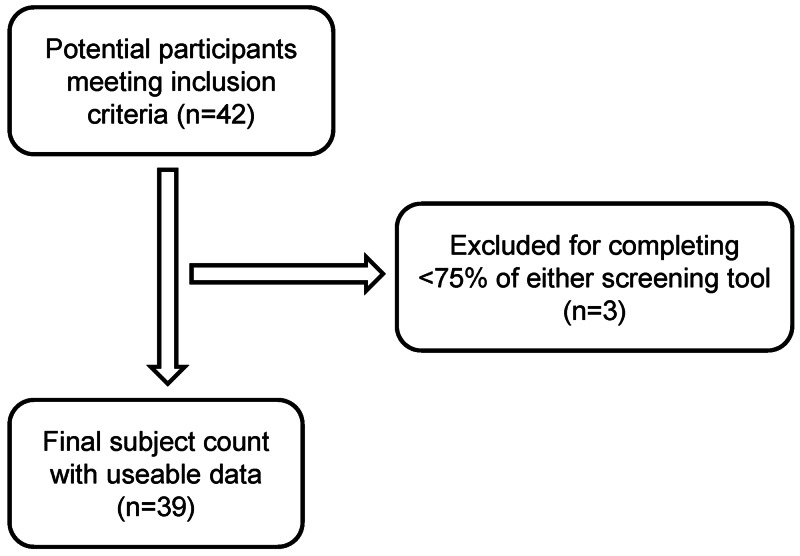
Subject inclusion algorithm

Study subjects included 42 female soccer players, ranging from 11 to 18 years old, who participated during one of four research study sessions. Subjects who completed less than 75% of the screening tool were not included, leading to a total of 39 subjects in the final pilot study population (Figure 1).

This pilot study received Emory University Institutional Review Board approval #92573. 

Prior to the administration of the screening tools to subjects, all packets were created. Packets included the RST and the PPGE. The investigators randomized screening tool order administration and the subjects were instructed to fill out the screening tools in the order given to them. Subjects were given unlimited time to complete the screening tool. 

Investigator A provided standardized instructions to the subjects. Investigators A and B provided verbal clarifications to subjects who had questions regarding survey items. Questions were permitted throughout the survey process. Investigator C collected and scored both screening tools following the session and was thus blinded to the participants.

The RST included subject age, height, and weight to allow for body mass index (BMI) calculation. A Likert scale was used for a majority of the screening tool questions to rate the risk of RED-S based on the subjects’ response (Appendix C). Scoring the screening tool for suspicion of RED-S is based on an extensive literature review and an expert pediatric orthopedic clinician’s experience with the syndrome.

## Results

Investigators calculated demographic data including BMI (mean 19.05, median 19.20, range 15.56-22.47) and age (11-18 years). Collection of participants' BMI relied on the subjective reports. 

Investigators calculated the total point score recorded on the RST (mean 185.6, median 159, range 52-452). Twenty-three percent of subjects were considered low risk and 77% of participants were considered moderate risk. Details for descriptive statistics for BMI, age, and each subsection are given in Table 1. 

**Table 1:** Pilot study RED-S descriptive statistics summary *Frequency for the menstrual function was determined by the number of subjects with primary amenorrhea. The frequency for injury was categorized as the number of subjects with a reported stress fracture. RED-S, Relative energy deficiency in sport; SD, Standard deviation; BMI, Body mass index; BMD, Bone mineral density; N/A, Not applicable

	Mean (SD)	Range	Median	Frequency*
BMI	19.05 (1.78)	15.58-22.47	19.2	N/A
Age	14.13 (1.99)	11-18	13	N/A
Menstrual function	3.21 (8.47)	0-25	N/A	0
Activity level	48.85 (26.34)	0-100	50	N/A
Nutrition/ Diet/ Weight	31.46 (31.30)	2-141	19	N/A
Injury	23.08 (42.68)	0-100	N/A	9
Factors affecting BMD	11.85 (13.36)	0-50	10	N/A
Psychological effects	17.23 (28.07)	0-75	50	N/A
Physiological effects	42.95 (17.57)	0-70	30	N/A
Total score	185.62 (95.61)	52-452	159	N/A

Using scoring criteria in Appendix A, investigators calculated the total point score recorded on the PPGE survey (mean 84.74, median 60, range 110-335). Of the subjects, 77% were considered low risk, 18% were considered moderate risk, and 5% of participants were considered high risk. Details for descriptive statistics for BMI, age, and each subsection can be found in Table 2. 

**Table 2:** PPGE survey descriptive statistics summary PPGE, Pre-Participation Gynecological Examination; SD, Standard deviation; BMI, Body mass index; N/A, Not applicable

	Mean (SD)	Range	Median
BMI	19.01 (1.80)	15.58-22.47	19.2
Age	14.13 (1.99)	11-18	13
Sports history	7.69 (15.34)	0-50	0
Menstrual antecedents	21.15 (38.70)	0-150	N/A
Influence of menstrual cycle on performance	10.26 (12.46)	0-25	0
Female athlete triad	37.31 (59.30)	10-185	10
Total score	84.74 (78.82)	110-335	60

Statistical package for the social sciences software (SPSS, IBM Corp., Armonk, NY) was used to perform concurrent validity analysis to validate the RST. A Pearson’s correlation was calculated to determine if there was a correlation between scores on the RST and the Triad-specific tool. Two independent t-tests were completed to determine if there was a difference between the means based on the administration order.

**Figure 2:**
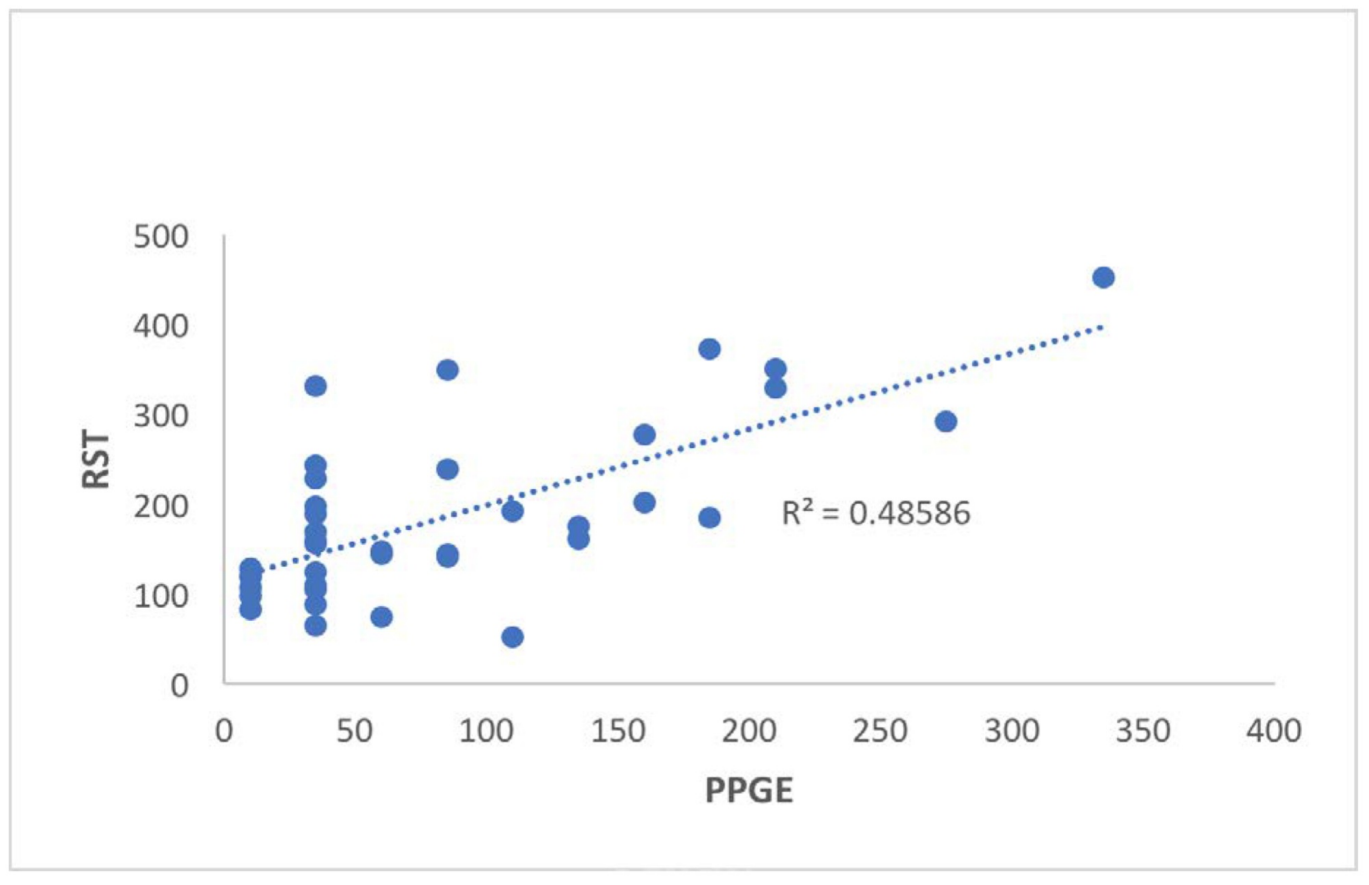
Correlation of the total point score between the RST and the PPGE The RST was validated against the PPGE. RED-S: Relative energy deficiency in sport; RST: RED-S-specific screening tool; PPGE: Pre-Participation Gynecological Examination

Pearson’s correlation between the PPGE survey and the RST showed a statistical significance between the two questionnaires (*r* = 0.697, *p* < 0.001) (Figure 2). It can be concluded that the RST was validated when compared with the PPGE survey. Randomness in administration order was validated based on an independent t-test. The order of administration of questionnaires had no significance on responses given (PPGE [*p* = 0.607], RST [*p* = 0.873]). 

## Discussion

It is imperative to raise awareness of screening opportunities and knowledge regarding low energy availability and its cost to the athlete. There is a need for an age-appropriate screening tool to assist groups such as athletes, coaches, and school health officials to identify conditions like the Triad and RED-S. While there have been validated screening tools for the Triad, a validated screening tool completed by adolescents is yet to be established for RED-S [15-16]. Mountjoy et al. published the RED-S clinical assessment tool (RED-S CAT) intended for clinician use [8]. Health benefits of screening and diagnosing RED-S in the young athlete include the prevention of sequelae of multisystem, long-term health problems, and allowing young patients to stay active throughout childhood, adolescence, and adulthood [3]. 

To identify RED-S in females who have yet to menstruate and males, it is helpful to utilize a screening tool that identifies other risk factors for these specific populations. Based on the pilot study, it can be concluded that there is a great need for education in the community about the Triad and RED-S. Furthermore, for the PPGE survey, there was a lack of diagnostic measures for females who have not yet menstruated. This is a vital distinction of the RST.

During the pilot study, researchers did allow the opportunity for subjects to ask questions if the subjects had difficulty comprehending the wording. Several words were modified like “modality” and one question regarding anemia was omitted secondary to confusion. Overall, it was noted that the middle-school females had more difficulty with the language of the PPGE survey questions than the RST questions, solidifying that the third-grade reading level was an accurate reading level for the young athletic population. It was notable that nine subjects identified that they had a stress fracture on the RST. In comparison, only seven subjects indicated that they previously had a stress fracture on the PPGE survey. This could possibly be due to more age-appropriate wording of questions on the RST. 

A few limitations were noted in this pilot study. The first limitation was that there is limited generalizability due to the small sample size of only 39 participants and the homogenous sample of soccer athletes used. The PPGE is not for males, but the EDS is used for males and females. Females were used for comparing because they could take both the PPGE and the RST. Secondly, subjects were allowed to ask questions while filling out the surveys, allowing for the collection of qualitative information. However, the questions asked were only answered on an individual basis. Not all participants chose to ask questions; however, we cannot assume that they did not have questions. Objective measurements were not obtained for BMI, rather BMI was calculated based on patient report of height and weight. For future clinical studies, height and weight should be objectively measured as this could potentially affect an individual’s final score. 

Future research may consider replicating this study using a different outcome measure other than the PPGE survey to increase the RST’s concurrent validity. Despite this tool’s limitations and the potential need for future validation in comparison to different tools, the RST acts as a beneficial tool when compared with the PPGE when screening young athletes for emerging signs of RED-S.

## Conclusions

The original hypothesis that the investigators would observe no significant difference between the means of the RST and the PPGE survey was statistically proven. The investigators, therefore, demonstrated the validity of the RST compared with the PPGE survey. This relationship was further strengthened when no significant difference was found between the means based on the order of administration. The RST may assist in age-appropriate screening for energy deficiency. This increase in knowledge and screening may lead to earlier identification and a more robust preventative approach to decreasing the risk of RED-S and the Triad in young athletes.

The goal of the RST is to be more comprehensive and sensitive in identifying risk factors for RED-S in male and female athletes. What the authors learned is the RST is also more age-appropriate. The readability allows the screening tool to be more friendly to the younger population and more widely accessible to the community. Utilization of the RST by coaches, athletic trainers, physical therapists, and other healthcare practitioners may increase knowledge and identification of the signs and symptoms related to the Triad and RED-S. Consequently, professionals may be able to detect RED-S risk and allow for earlier intervention.

## Figures and Tables

**Figure 3:**
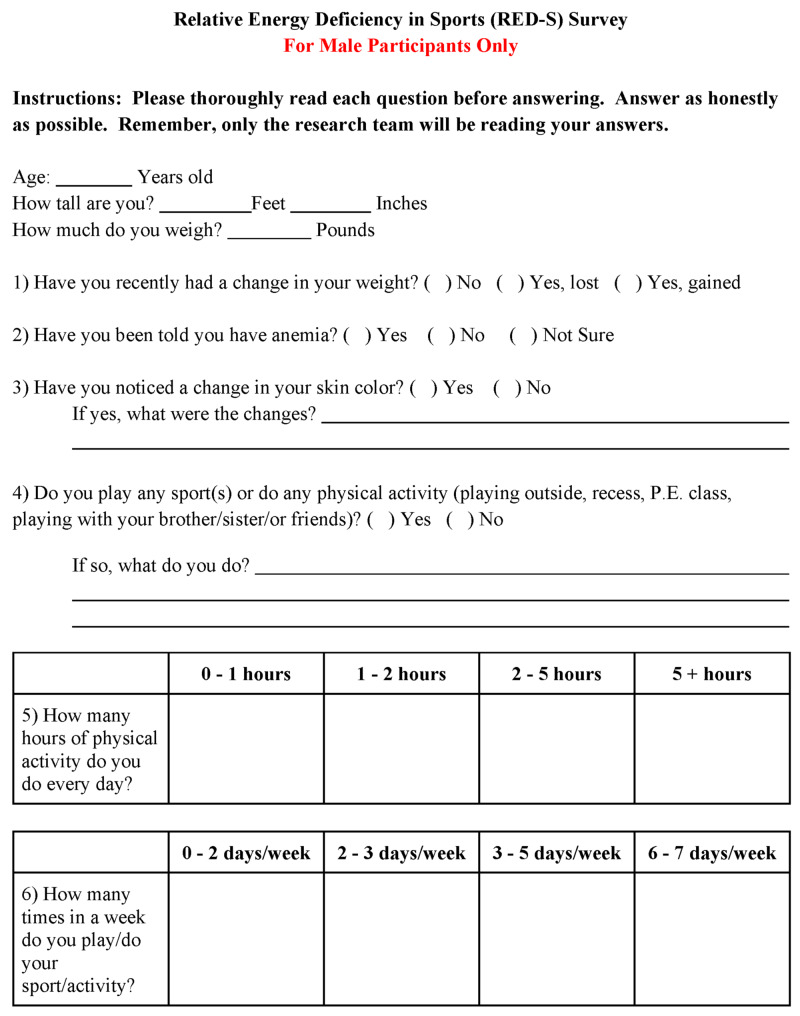
Male RED-S screening tool RED-S, relative energy deficiency in sport

**Figure 4:**
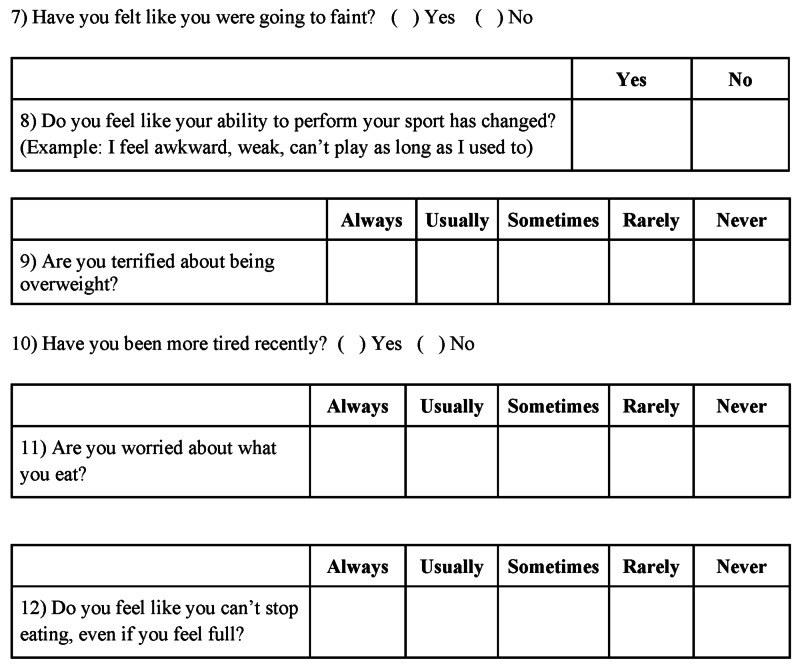
Male RED-S screening tool RED-S, relative energy deficiency in sport

**Figure 5:**
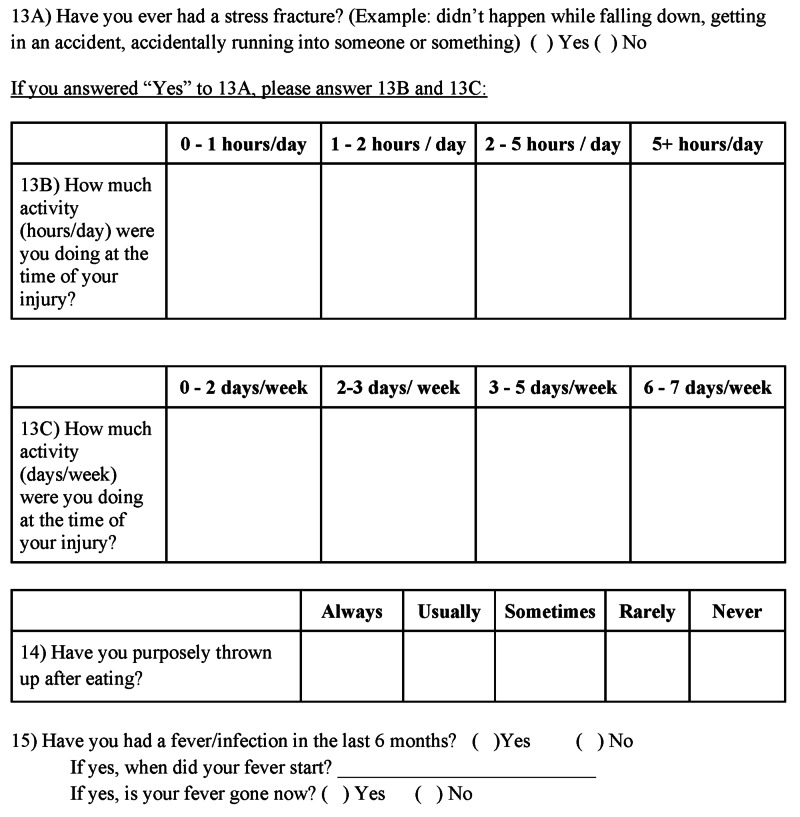
Male RED-S screening tool RED-S, relative energy deficiency in sport

**Figure 6:**
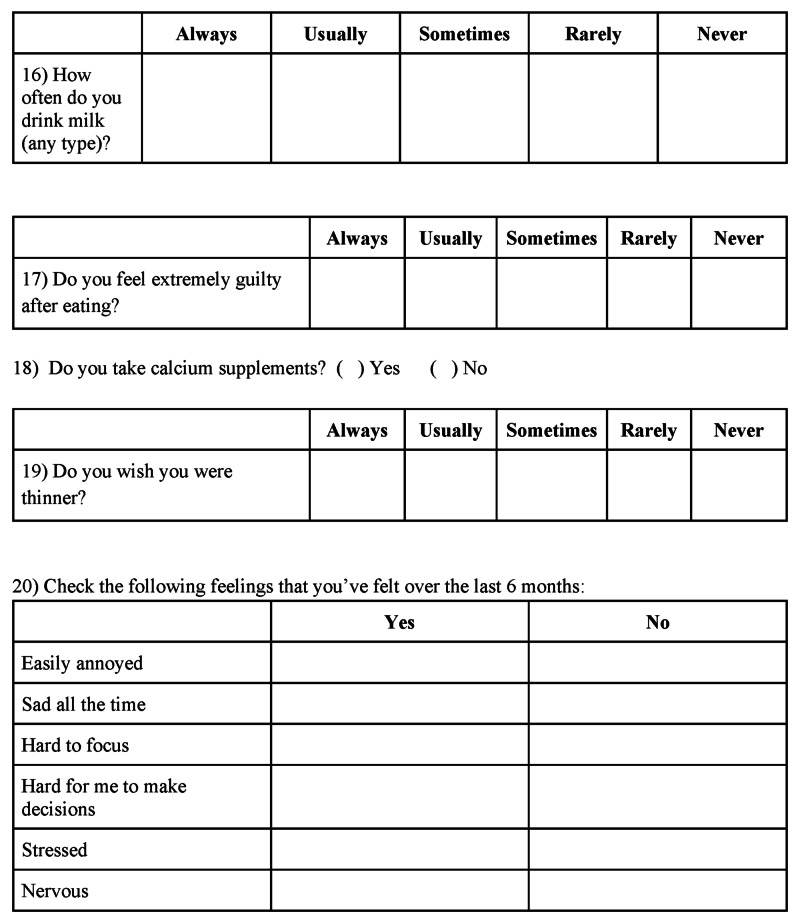
Male RED-S screening tool RED-S, relative energy deficiency in sport

**Figure 7:**
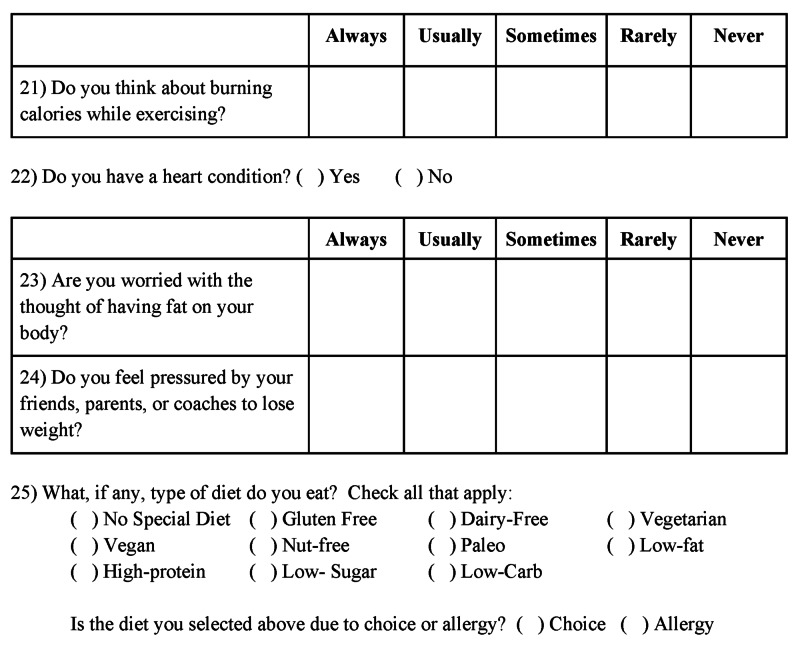
Male RED-S screening tool RED-S, relative energy deficiency in sport

**Figure 8:**
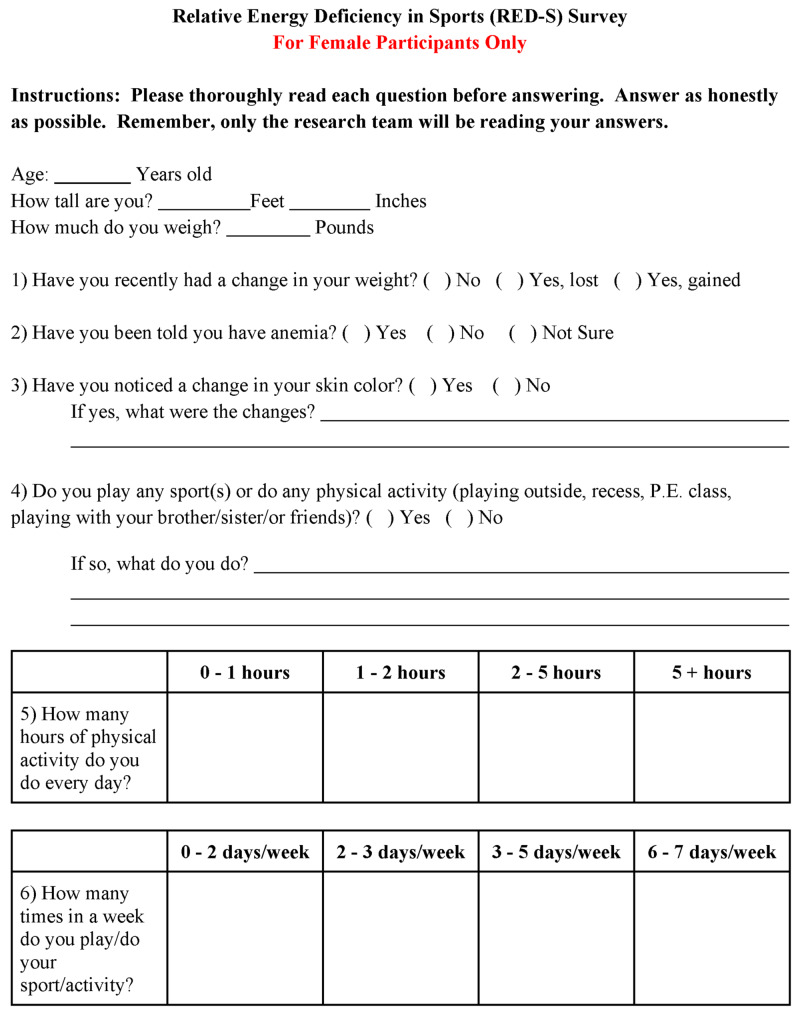
Female RED-S screening tool RED-S, relative energy deficiency in sport

**Figure 9:**
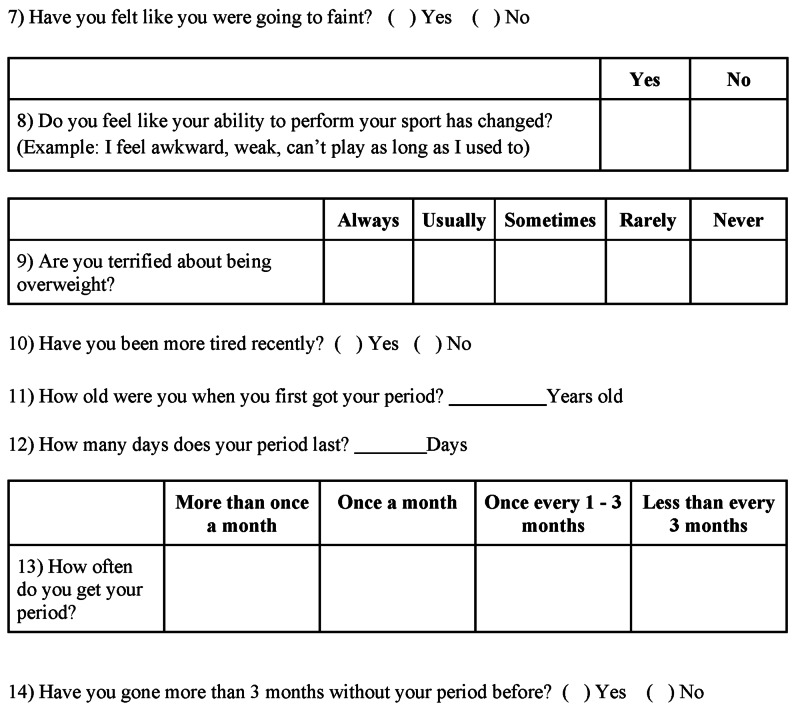
Female RED-S screening tool RED-S, relative energy deficiency in sport

**Figure 10:**
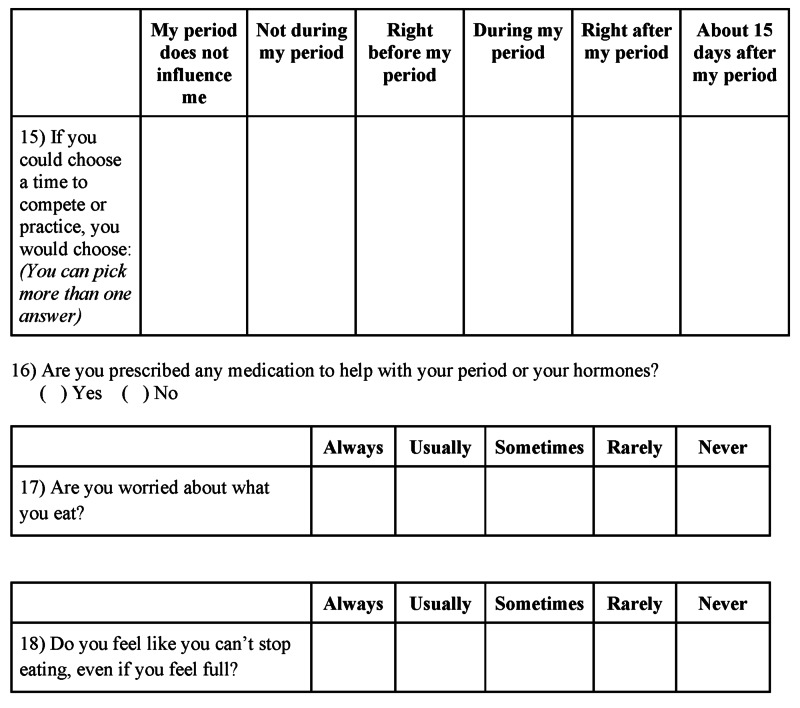
Female RED-S screening tool RED-S, relative energy deficiency in sport

**Figure 11:**
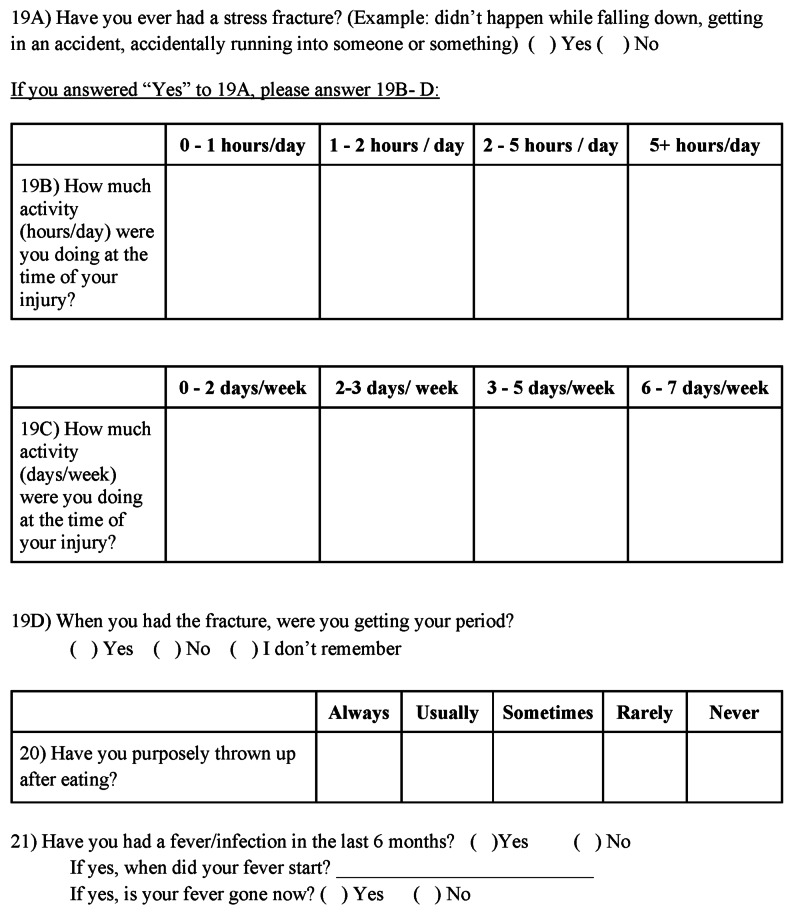
Female RED-S screening tool RED-S, relative energy deficiency in sport

**Figure 12:**
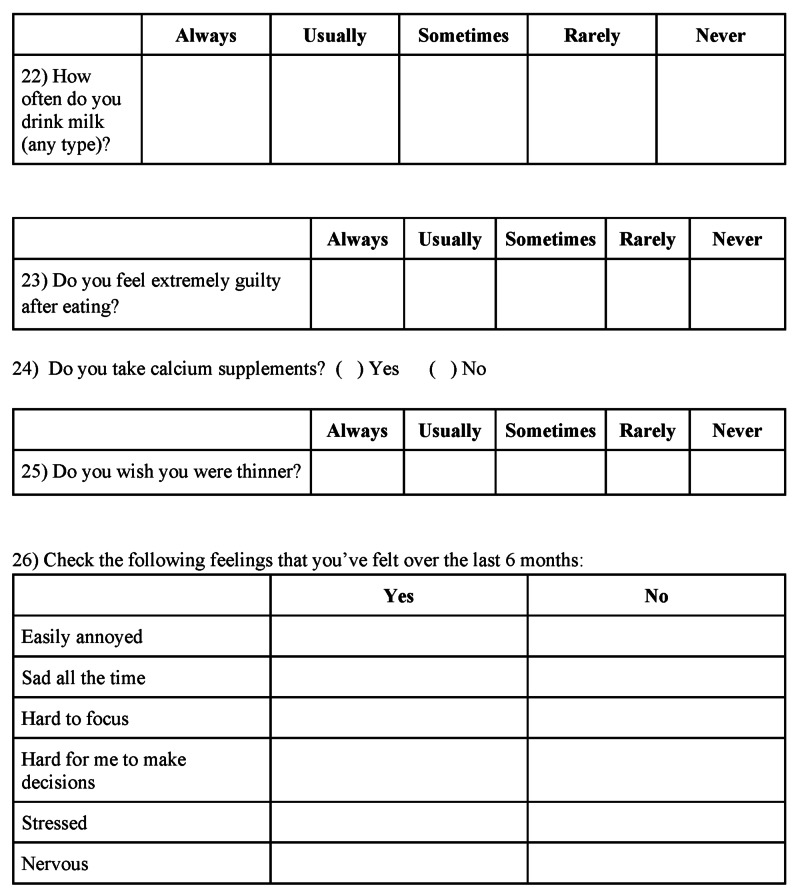
Female RED-S screening tool RED-S, relative energy deficiency in sport

**Figure 13:**
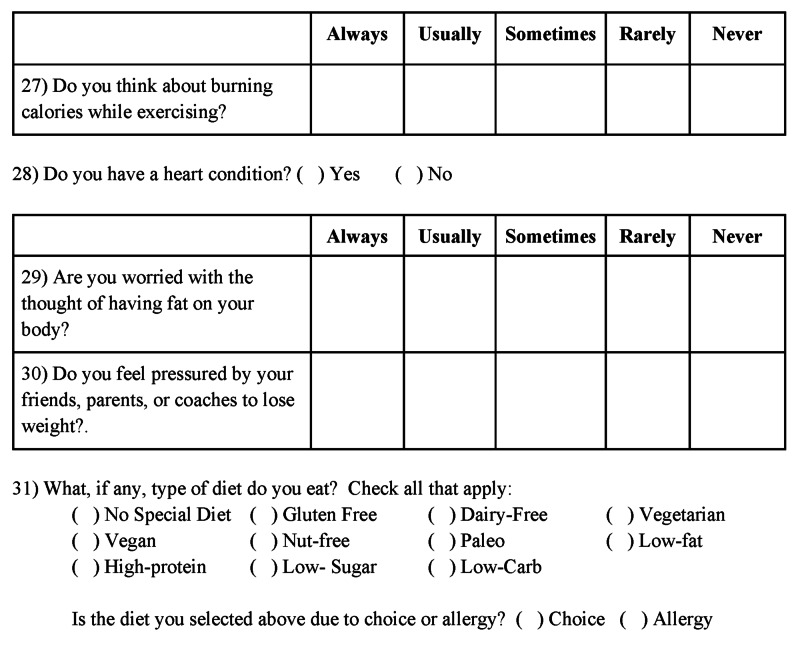
Female RED-S screening tool RED-S, relative energy deficiency in sport

## Appendices

**Appendix A. Scoring Appendix for the Pre-Participation Gynecological Examination (PPGE) Survey** For the PPGE survey, the total possible score for menstruating female participants was 710 points. For this group, a score of 0-150 points was categorized as low risk, between 151 and 300 points as moderate risk, and above 300 points as high risk. The total possible score for female participants who had yet to menstruate and/or were younger than 16 years of age was 310 points. For this group, a score of 0-75 points was considered low risk, 76-150 points as moderate risk, and greater than 150 points as high risk (Appendix A Table 3). The PPGE survey was subdivided into the following categories: general data, sports history, menstrual antecedents, the influence of menstrual cycle on performance, premenstrual syndrome (PMS), female athlete triad, and past medical and surgical issues. The PMS section was not quantitatively scored (Appendix A Table 4). 

**Table 3:** Pilot Study PPGE Survey Scoring Classifications to determine the risk level PPGE, Pre-Participation Gynecological Examination

Classification	Females <16 years	Females ≥16 years
Low risk	0-75	0-150
Moderate risk	76-150	151-300
High risk	151-310	301-710
Maximum possible score	310	710

**Table 4:** Pilot Study PPGE Survey Scoring Rubric Per Subsection PPGE, Pre-Participation Gynecological Examination

Subsection of PPGE survey	Maximum possible score of subsection
General data	25 (0 for females < 16 not menstruating)
Sports history	50
Menstrual antecedents	325 (0 for females < 16 not menstruating)
Influence of menstrual cycle on performance	50 (0 for females < 16 not menstruating)
Premenstrual syndrome	0 (Informational)
Female athlete triad	235
Past medical and surgical issues	25

**Appendix B. Scoring Appendix for Relative Energy Deficiency in Sport (RED-S) Screening Tool** For the RED-S screening tool (RST), the total possible score for menstruating female participants was 880 points. For this group, a score of 0-150 points was categorized as low risk, between 151-500 points as moderate risk, and above 500 points as high risk. The total possible score for female participants who had yet to menstruate and/or were younger than 16 years of age was 730 points. For this group, a score of 0-100 points was considered low risk, 101-400 points as moderate risk, and greater than 400 points as high risk (Appendix B Table 5). The RST was subdivided into the following categories: menstrual function, activity level, nutrition/diet/weight, injury, factors affecting bone mineral density, psychological effects, and physiologic events. The maximum possible total for each subsection is listed in Appendix B Table 6. 

**Table 5:** Pilot Study RED-S Scoring Classifications to determine the risk level RED-S, relative energy deficiency in sport

	Females ≥ 16 years old and menstruating females ≤ 16 years old	Females without onset of menarche and/or < 16 years old
Low risk	0-100	0-150
Moderate risk	101-400	151-500
High risk	> 400	> 500
Maximum possible score	880	730

**Table 6:** Pilot Study RED-S Scoring Rubric Per Subsection RED-S, relative energy deficiency in sport

Subsection of RED-S Screening Tool	Maximum Possible Score of Subsection
Menstrual function	150 (0 for females under 16 not menstruating)
Activity levels	100
Nutrition/diet/weight	290
Injury	100
Factors that affect bone mineral density	50
Psychologic effects	75
Physiological effects	115

Appendix C. Relative Energy Deficiency in Sport (RED-S) Screening Tools 
